# Glioma Stem Cells and Their Microenvironments: Providers of Challenging Therapeutic Targets

**DOI:** 10.1155/2016/5728438

**Published:** 2016-02-10

**Authors:** Elena Codrici, Ana-Maria Enciu, Ionela-Daniela Popescu, Simona Mihai, Cristiana Tanase

**Affiliations:** ^1^Biochemistry-Proteomics Department, Victor Babes National Institute of Pathology, Sector 5, 050096 Bucharest, Romania; ^2^Cell Biology and Histology Department, Carol Davila University of Medicine and Pharmacy, Sector 5, 050474 Bucharest, Romania

## Abstract

Malignant gliomas are aggressive brain tumors with limited therapeutic options, possibly because of highly tumorigenic subpopulations of glioma stem cells. These cells require specific microenvironments to maintain their “stemness,” described as perivascular and hypoxic niches. Each of those niches induces particular signatures in glioma stem cells (e.g., activation of Notch signaling, secretion of VEGF, bFGF, SDF1 for the vascular niche, activation of HIF2*α*, and metabolic reprogramming for hypoxic niche). Recently, accumulated knowledge on tumor-associated macrophages, possibly delineating a third niche, has underlined the role of immune cells in glioma progression,* via* specific chemoattractant factors and cytokines, such as macrophage-colony stimulation factor (M-CSF). The local or myeloid origin of this new component of glioma stem cells niche is yet to be determined. Such niches are being increasingly recognized as key regulators involved in multiple stages of disease progression, therapy resistance, immune-escaping, and distant metastasis, thereby substantially impacting the future development of frontline interventions in clinical oncology. This review focuses on the microenvironment impact on the glioma stem cell biology, emphasizing GSCs cross talk with hypoxic, perivascular, and immune niches and their potential use as targeted therapy.

## 1. Introduction

Gliomas, representing tumors of astroglial origin, have been classified by World Health Organization (WHO) into four grades of ascending malignancy according to the histological criteria. Presenting one of the highest mortality rates, glioblastoma multiforme (GBM, WHO grade IV) only benefits from palliation as far as conventional therapy goes. In spite of intensive efforts and the progress achieved in tumor biology and clinical treatment, little improvement of the average survival for a newly diagnosed GBM patient to less than 15 months was recorded [1]. Both GBM biology in general and the cellular origin of this disease in particular are not fully understood, thus restraining clinical advances. Vascular endothelial proliferation appears to be a highly angiogenic tumor in GBM, since extensive blood vessel growth is essential for tumor progression and invasion [2]. The vasculature is associated with GBM, reducing hypoxia; it is generally required for tumor survival. The cancer stem cell hypothesis suggests that all cancer types are comprised of a subset of highly aggressive cells. These propagate and preserve the tumors thought to have unlimited self-renewal capacity and potent tumorigenicity [3].

GSCs and normal neural stem cells (NSCs) present similar properties, such as the expression of neural stem cell markers, infinite self-renewal and long-term proliferation ability, neurospheres formation, and multipotential differentiation capacity [3, 4]. Furthermore, according to* in vivo* evidence, GSCs can initiate highly invasive tumors [5]. GSCs have been proven to be resistant to various chemotherapeutic agents, such as temozolomide, the standard chemotherapeutic agent for GBM treatment, allowing these cells to survive therapy, leading to disease recurrence [6–8].

It has been recently demonstrated by experimental studies that GSCs are enriched in specific niches around tumor vessels and areas of necrosis, the latter associated with restricted oxygen levels. Hence, GSCs display a symbiotic relationship with perivascular/proliferative and hypoxic/perinecrotic niches [8–10]. Endothelial cells (ECs) generate numerous growth factors that fuel GSC self-renewal, tumorigenicity, and survival [11–13]. GSCs may transdifferentiate into endothelial cells or pericytes, forming their own vascular niches [14–17]. The capacity of GSCs to transdifferentiate into functional endothelial cells is still under debate. While conventional theory suggests that GBM tumor vasculature derives from existing vessels or from bone marrow progenitor cells, there are recent studies that sustain the hypothesis that a large subset of endothelial cells can be generated by GSCs [18].

Many cytokines and chemokines are produced by GSCs as well, some of which are known to activate endothelial cells [19–21]. According to this hypothesis, GSCs may in turn regulate the tumor vasculature and, consequently, the extent of tumor angiogenesis.

The main focus of this review will be on the interaction between GSCs and their microenvironment, emphasizing the molecular processes through which GSCs cross-talk with hypoxic and vascular niches. Another key point will be the interaction of GSCs with tumor-infiltrating immune cells and the role of GSCs in the regulation of tumor angiogenesis in GBM.

## 2. Glioma Stem Cells (GSCs) and Their Markers

Infinite self-renewal, unlimited proliferative potential, multilineage differentiation capacity, neurospheres formation, and expression of neural stem cell markers (e.g., CD133/prominin-1, Sox2, and Nestin) represent some of the specific features of GSCs [22, 23]. Other various candidate markers that are used in order to enrich GSCs have been discovered over the last ten years, among which are CD44 [24], CD49f (integrin a6) [25], Musashi [26], Nestin [27, 28], Nanog [29–31], Oct4 [29, 30, 32], and Sox2 [33, 34]; nevertheless, the quest for a universal GSCs marker continues [4].

GSCs seem to be genuine cancer reservoirs; consequently, any therapy approach aiming at brain cancers is obstructed by the resistance to treatment that these cells show, since GSCs are capable of whole tumor regeneration once the treatment has concluded [35, 36].

Lathia et al. demonstrated that GSCs constitute the origin and source of tumor recurrence in glioblastoma [7, 8], by injecting differentially labeled GSCs and non-GSCs into mice. The only fraction to produce tumors was the GSC, despite representing only 10% of the implanted cells [8]. It appears that the ones responsible for tumor growth are GSCs, rather than the more differentiated tumor cells. In addition, the former are also involved in tumor recurrence following drug resistance. Chen et al. showed that when temozolomide treatment is interrupted in a spontaneous murine glioma model, Nestin-positive GSC population is the first cell population that undergoes proliferation and leads to tumor regrowth [7]. GSCs have been found to be enriched in recurrent gliomas [37, 38]. When isolating GSCs from recurrent tumors, they generate more aggressive invasive tumors in athymic mice than when isolated from primary tumors derived from the same patient [38]. Subsequently, it seems that GSCs contribute to tumor regrowth from minimal residual disease after surgery. GSCs display great resistance to chemotherapeutic agents, as well as a highly invasive feature [39]. Another property of GSCs in terms of resistance is their particular resistance to radiation, in comparison with the more differentiated glioma cells, with this being the consequence of an effective DNA damage repair response [35]. Notch signaling in GSCs promotes self-renewal, protects against radiation, and represses differentiation [40].

Research has focused particularly on the identification of intrinsic molecular pathways involved in the regulation of GSCs features, such as stemness and tumorigenicity, ever since the GSCs have been identified [5, 41, 42] (Table 1).

### 2.1. CD133 Controversy

The identification of specific surface markers is necessary in order to isolate GSCs and subsequently characterize them for future GSC-targeted therapies [90]. CD133 (prominin-1) is one of the earliest stem-cell surface markers used for identification and isolation of cancer stem cells in malignant brain tumors. Singh et al. successfully isolated a CD133+ cell subpopulation from human brain tumors that exhibited stem cell properties* in vitro*. They reported the development of a xenograft that identified human brain tumor initiating cells that initiate tumors* in vivo*. Only the CD133+ brain tumor cells could initiate tumor in mouse brain, whereas injection of CD133− cells did not lead to tumor formation [22].

However, accumulated results in GBM molecular research led to several CD133 related controversies. For example, GSCs display a variation in the levels of CD133 expression that did not directly correlate with the tumorigenic potential [91]. Most importantly, different studies suggested that CD133− tumor cells isolated from GBMs can also be stably cultured under stem cell conditions. Similar to the CD133+ cells, these cells also showed “stem cell” properties such as self-renewal, differentiation* in vitro*, and formed transplantable tumors in a xenograft model [92, 93]. Further phenotypic analysis showed that unlike the CD133+ cells, which can form floating spheroids in culture, the CD133− cells tend to grow as adherent spheres. This observation led to the assumption that CD133+ and CD133− cells may originate from different pools of self-renewing glioma stem cells (GSCs) [94]. It has recently been reported that a small population of CD133− cells can give rise to CD133+ cells, suggesting a possible stem cell hierarchy in the spheroid culture system that may or may not have* in vivo* relevance [95]. These results, however, have been brought into discussion in 2013 by Brescia et al., who argued that the + or − CD133 status depends, in fact, on the protein subcellular localization between the cytoplasm and the plasma membrane [96].

Data continues to accumulate, however, on CD133 biology, as it has been repeatedly demonstrated to be essential for GSC maintenance and neurosphere formation [96] and it is a good indicator of resistance to conventional therapies [36].

Moreover, the association of CD133 with other markers could enhance the potential pathological prognostic markers for glioma patients [97]. For instance, the association of coexpression of Nestin/CD133 is helpful in predicting the aggressive nature of gliomas [28]. The presence of CD133+/Ki67+ positive cells may be an indicator of tumor progression and unfavorable prognosis [98].

### 2.2. Intratumor Heterogeneity of GSCs

Yet another factor to be added to the difficult task of GSCs characterization is the heterogeneity of their own population. At least two phenotypes of GSCs (proneural and mesenchymal phenotypes) have been reported [99], each characterized by a different transcriptional profile [100] and different metabolism [101].

Single cell-derived clones of human glioblastoma tumors with stem properties (e.g., able to reconstitute the original tumor) exhibited functional and morphologic heterogeneity. Even though* in vitro* all clones displayed neuronal precursor phenotype, individual clone-derived populations expressed different GBM markers (such as EGFR, EGFRvIII, and PTEN) and clone by clone variability in response to multiple drugs [102].

Further on, heterogeneity arises with microenvironment change. If* in vitro* nonproliferating and proliferating cells of the parental tumor showed no significant differences in their transcriptional profiles,* in vivo* clonal orthotopic tumors derived from proliferative cells upregulated distinct sets of genes, when compared with their nonproliferative counterparts [103].

A functional consequence of transcriptional and metabolic heterogeneity is the frequency of self-renewal and differentiation rate of progenitor cells. The question whether GSC heterogeneity is maintained during repetitive cycles of self-renewal or lost to those clones with high frequency cell division has been recently answered by Sugimori et al. They reported that “the growth characteristics of GSs are retained during repopulation … and do not support the clonal evolution model, at least not with regard to SC heterogeneity.” It seems that, in order to recapitulate over generations, the heterogeneity of the initial population, at least in terms of proliferative activity, cancer stem cells must exhibit plasticity, meaning that “clones change their spatial and temporal properties” [104].

## 3. GSCs and Their Microenvironment (Niches)

Stem cells and these niches do not display a passive relationship; they have a dynamical interaction with their microenvironment. While stem cells actively influence their microenvironments, they are regulated by signaling from that same microenvironment. Likewise, GSCs also exist in specific niches that play a role in enhancing the stem-like features of GSCs, promote invasion and metastasis of GSCs, and even affect response to therapy/escape from therapy. It is essential to understand the bidirectional cross talk between GCSs and the niches in order to disclose the role of this controversial population in GBM initiation, progression, invasion, and therapeutic resistance.

### 3.1. The Perivascular/Proliferative Niche

In perivascular regions, GSCs appear to be enriched, where a great deal of regional signals have been found to promote their phenotypes [105]. GSCs are generally located near the endothelial cells (ECs) that line capillaries, especially in the subventricular zone and the hippocampus [106, 107].

#### 3.1.1. Components of Vascular Niche

*(1) Soluble Factors: Origin and Effect on GSCs*. It has been reported that GSCs release high levels of proangiogenic factors, such as vascular endothelial growth factor (VEGF) that drives the migration of newly EC into the mass and promotes angiogenesis. ECs overexpress VEGF receptors (VEGFR2); thus, an environment of high VEGF increased ECs proliferation, migration, and blood vessel permeability. Permeability alterations are associated with increased edema usually observed in GBM [106].

Moreover, Sonic Hedgehog (SHH) is considered one of the central soluble factors secreted by ECs that promote the acquirement of CSC properties by activating the HH signaling pathway. GSCs display active SHH-GLI1 signaling and regulate GSC self-renewal and glioma growth [46, 108]. In primary glioma samples, GSCs CD133+ are found in the area near SHH-expressing ECs. Tumor sphere formation and the expression of stemness-related molecules are promoted by ECs through glioma associated oncogene homologue 1 (GLI1) enhancement and its translocation from the cytoplasm to the nucleus [109].

Tumor ECs expressed SHH [110] in a PDGF-driven mouse glioma model, providing a potential mechanism for GLI1 activation in GSCs.

It has been recently found that the secretion of FGF-2 by GBM cells enhances the blood brain barrier function of ECs, which also contributes to drug resistance in GBM [111]. Survivin, an angiogenesis-promoting protein, could activate the release of FGF-2, along with VEGF, in gliomas and thereby stimulate an increase in growth and proliferation in the tumors [112]. FGF-2 helps maintain especially GSCs stemness. When removed from GSCs lines, it resulted in differentiation; this was not observed when the cells were in the presence of the growth factor [113]. FGF-2 is effective at inducing Nestin, in C6 glioma cells, proving its contribution to the stemness of glioma cells [114]. Autocrine production of FGF-2 in combination with EGF may also be responsible for retaining the self-renewal potential of GSCs [115]. FGF-2's role in GSCs remains to be characterized. The therapies targeting FGF-2 might be effective at destroying GSCs, since the growth factor is important in preserving the stemness feature of GSCs [116].

Osteopontin, which is derived from the perivascular niche, promotes GSCs phenotype by activating CD44, one of the CSC markers. The C-terminal intracellular domain of CD44 is essential for inducing GSCs characteristics by enhancing the function of hypoxia inducible factor 2*α* (HIF-2*α*) [117].

Besides the above-mentioned factors, GSCs secrete other proangiogenic growth factors as well. When comparing the proteomes of four different GSCs with four normal NSCs cultures, the levels of HDGF (hepatoma-derived growth factor) were found twofold higher in GSCs. By further analyzing the GSC conditioned medium, it has been revealed that only GSCs secrete HDGF, which promotes endothelial cell migration* in vitro* and angiogenesis in a subcutaneous* in vivo* model [21]. Identifying a specific angiogenic factor by GSCs as compared to normal stem cells allows selective targeting of tumor angiogenesis without affecting the normal stem cell pool. This proves particularly significant data, suggesting that normal stem cells produce cytokines (e.g., BMP7), which act as suppressors of GSC activity [118].

An essential growth factor expressed in GBM is growth hormone releasing hormone (GHRH) [119]. It causes increased tumor cell proliferation, migration, and tumor progression. GHRH may also play a role in the activation of stromal fibroblasts in the tumor microenvironment by regulating *α*-SMA expression. It remains to be elucidated whether GHRH specifically affects GSCs and its effects on tumor endothelial cells [120].

**Figure 1:**
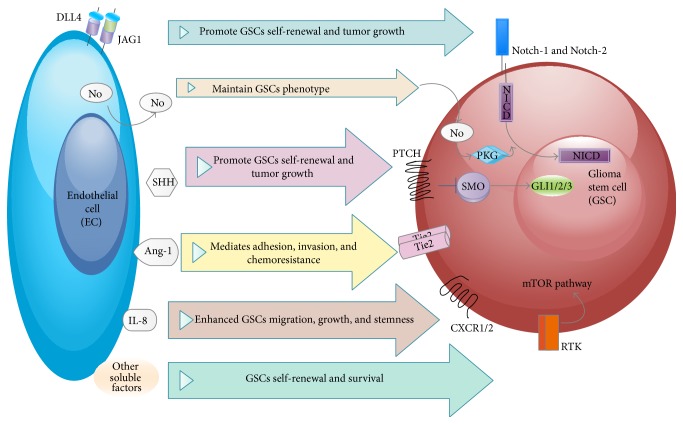
*Effects of endothelial cells on GSCs. ECs produce membrane-bound Notch ligands* Jagged-1 (JAG1) and Delta-like 4 (DLL4) that bind to Notch receptors on GSCs and promote GSCs self-renewal and tumor growth;* nitric oxide* (NO) that maintains GSCs phenotype;* ligand sonic hedgehog* (SHH) that promotes GSCs self-renewal and tumor growth;* angiopoietin-1* (Ang-1) that mediates adhesion, invasion, and chemoresistance;* IL-8* that enhanced GSCs migration, growth, and stemness;* other soluble factors* that stimulate GSCs self-renewal and survival.

*(2) Cells: Cell to Cell Interactions*. The interaction between GSCs and ECs promotes activity in critical stem pathways, such as Notch signaling. GSCs Nestin-positive cells express the Notch receptors Notch-1 and Notch-2 and show elevated level of Notch activity [11]. ECs express the Notch ligands Delta-like 4 (DLL4) and Jagged-1. Knockdown of these ligands in brain microvascular endothelial cells (BMECs) reduced tumor growth upon cotransplantation of GSCs with BMECs [11]. GSCs may directly stimulate the expression of Notch ligands on ECs suggested by the findings that GSCs secrete elevated levels of VEGF [19], which induces DLL4 expression in ECs [121, 122] (Figure 1).

EC-derived nitric oxide (NO) can activate Notch signaling pathway in GSCs as well through NO/cGMP/PKG; therefore, it promotes the stem cell phenotype [123, 124]. GSCs produce NO endogenously, which supports GSC growth and tumorigenicity [124]. Endothelial nitric oxide synthase (eNOS) also produces NO in the tumor vasculature. Upon loss of eNOS, it suppresses Notch signaling* in vivo*, it delays glioma genesis, and it prolongs the survival of tumor-bearing mice [123].

Furthermore, combined treatment of CSCs and vascular niches should not be overseen. Following radiation, Jagged-1, the ligand for Notch, was shown to be increased in ECs [125]. This suggests that Notch signaling is critical for EC-mediated radioresistance of CSCs [126].

*(3) Extracellular Matrix*. The perivascular region is also enriched for extracellular matrix proteins (e.g., laminin) that are capable of promoting proliferation, survival, and migration of GSCs. GSCs are enriched for integrin *α*6 [25], acting as a receptor for laminin in complex with integrin *β*1 or *β*4. The integrin *α*6-expressing cell population is localized in the perivascular compartment of human GBM and silencing of integrin *α*6 reduces the self-renewal and tumorigenicity of glioblastoma cells. Likewise, adult NSCs, which are closely apposed to the laminin-containing extracellular matrix surrounding vascular endothelial cells, express *α*6/*β*1 integrin and its blockade inhibits neural stem cell adhesion to endothelial cells [127]. Integrins *α*6-*β*1 also play a cytoprotective role for ECs by increasing expression of antiapoptotic proteins, such as cFLIP, and inducing the prosurvival of the TNF*α* pathway [128].

The interaction between vascular niche and GSCs also involves chemokines and their receptors. CXCR4 works as a biomarker of CSCs in several types of cancer, including glioma [129]. CXCR4-positive tumor cells can self-renew in a serum-free medium and display potent tumor-initiating capability. The ligand for CXCR4, namely, CXCL12, is secreted by ECs and the immune cells in tumor microenvironment [130], which highlights the importance of CXCL12/CXCR4 axis in the maintenance of GSCs in vascular niches. By using a three-dimensional culture system, Infanger et al. proved that ECs promoted GSC-like properties by secreting enhanced levels of the chemokine CXCL8/IL-8 and upregulating its cognate receptors CXCR1 and CXCR2 [12]. According to these results, chemokine signaling is involved in vascular niches stemness regulation of GSCs.

#### 3.1.2. The Role of Perivascular Niche in GSCs Biology

Perivascular niche appears to play a role in promoting the radioresistance of brain tumor CSCs. Due to their ability to activate the PI3K/Akt/mTOR pathway and undergo transient, PTEN and p53-dependent cell cycle arrest, CSCs localized in the vicinity of blood vessels in the brain were resistant to radiation. Inhibition of Akt signaling sensitized perivascular CSCs to radiation-induced apoptosis [131]. Inhibition of Notch signaling with gamma-secretase inhibitors (GSIs) impaired radiation-induced Akt activation and increased radiosensitivity of glioma stem cells. Knockdown of Notch-1 or Notch-2 sensitized glioma stem cells to radiation. The radioprotective functions of Notch were specific for GSCs but not non-GSCs [40].

ECs of vascular niches are crucial for inducing chemotherapy resistance of GSCs. Nonetheless, according to recent studies, mural cells of vascular niches also played a role in the induction of drug resistance of GSCs. The protective role of ECs and mural cells in GSCs resistance against radio-/chemotherapy emphasizes the importance of vascular niche in targeted cancer therapy.

#### 3.1.3. GSCs Can Shape the Perivascular Niche

GSC-derived factor stimulates the ECs proliferation, angiogenesis; GSCs recruit endothelial progenitor cells from bone marrow and GSCs transdifferentiation into pericytes.

Pericyte recruitment is induced by ECs release signals [132]. Pericytes secrete growth factors that stimulate ECs proliferation and proteases that contribute to the modulation of the surrounding extracellular matrix and guide ECs migration [133]. The resulting pericyte coverage is crucial for vessel remodeling, maturation, and stabilization and has been involved in therapeutic resistance in tumors. Direct contact establishes reciprocal communication between ECs and pericytes, either by paracrine signaling or by a newly described chemomechanical signaling pathway [134]. Signaling molecules such as angiopoietin-1/2 and Tie2 (Ang/Tie2), transforming growth factor-*β* (TGF-*β*/TGF-*β*R), and platelet-derived growth factor-*β* (PDGF*β*/PDGFR-*β*), which are related to EC viability, mural cell differentiation, and pericyte recruitment, respectively, are involved in the cross talk coordination [135].

Based on the work of Ricci-Vitiani et al., it appears that part of the vasculature in GBM originates from tumor cells. They analyzed the vasculature in 15 human glioblastoma patient samples and found that a large subset of endothelial cells harbored the same mutations and chromosomal aberrations as the tumors themselves. They also showed that* in vitro* culture of GSCs in endothelial conditions generated progeny with phenotypic and functional features of endothelial cells. Subcutaneous injection of GSCs in immunocompromised mice produced tumor xenografts; the tumor vessels were composed of human endothelial cells. All these findings describe a new mechanism for tumor vasculogenesis and may explain the presence of cancer-derived endothelial-like cells in several malignancies [15].

Various other studies have recently explored the phenomenon of tumor-derived vasculature in GBM. Recent reports found very similar results to those of Ricci-Vitiani et al., showing that oncogene induced glioblastoma tumors gave rise to tumor-derived endothelial cells, as indicated by GFP expression. These studies also found that a subpopulation of endothelial cells within tumors harbored the same genetic signature as the tumor itself [14, 136]. Chiao et al. reported that GCSs formed vasculogenic mimicry in tumor xenografts and expressed provascular molecules [137].

However, Rodriguez et al. mention that “while the potential of stem-like cancer cells to form endothelium in culture seems clear, in our clinical experience using a variety of molecular markers, neoplastic cells do not contribute significantly to the endothelial-lined vasculature of primary human glioblastoma.” At the end of the study, their observations were that glioblastoma cells incorporated into tumor vessels appear rather unfrequent, and thus it is of questionable clinical or therapeutic significance [138].

Interestingly, Cheng et al. present an alternative hypothesis to that of Ricci-Vitiani et al. by showing that GSCs can give rise to vascular pericytes (that may actively remodel perivascular niches) which also express Tie2, rather than endothelial cells. Targeting these GSC-derived pericytes disrupted vessel function and inhibited tumor size similarly as the results presented by Ricci-Vitiani et al. for targeting endothelial cells [16]. El Hallani et al. suggested that rather than transdifferentiating, the GSCs were fusing with endothelial cells to create a hybrid tumor vasculature [139, 140]. Conversely, using a GSC mouse xenograft, Lathia et al. did not mention the integration of tumor-derived cells into the vascular wall [8].

Zheng et al. found that, unlike circulating EPCs, the endothelial progenitor cells (EPCs) present in the tumor tissues share genetic aberrations with the tumor cells. The presence of genetic aberrations of glioma cells (EGFR amplification, PTEN deletion, and aneusomy of chromosomes 7 and 10) in intratumoral EPCs may point to transdifferentiation of GSCs into EPCs [141].

It has recently been suggested that GSCs localized near perivascular niches promote angiogenesis in GBM, possibly through differentiation into ECs. Alternatively, GSCs can undergo mesenchymal differentiation and may differentiate into tumor pericytes [142]. Cheng et al. showed that most pericytes were derived from neoplastic cells in human GBM specimens by combined analyses of common genetic changes and the expression of pericyte marker including *α*-smooth muscle actin (*α*-SMA), NG2, and platelet-derived growth factor receptor (PDGFR),* in vitro* [16, 17]. By means of lineage-tracing analysis* in vivo*, they showed that GSCs gave rise to the majority of vascular pericytes in GBM xenografts but did not generate tumor ECs. These GSC-derived cells expressed a panel of pericyte markers; however, they no longer expressed putative GSC markers, indicative of commitment to the pericyte lineage. Pericytes isolated from primary human GBMs or xenografts harbored the same genetic alterations as matched GSCs, suggesting that vascular pericytes predominantly derive from neoplastic cells. TGF-*β* signaling from ECs induced differentiation of GSCs into pericytes, at least in part; it was dependent on recruitment by EC-secreted stromal cell-derived factor 1 (SDF-1), which signaled through CXCR4 expressed on GSCs. Selective elimination/deletion of GSC-derived pericytes in tumor-bearing mice disrupted tumor vessel structure and impaired vascular function, resulting in inhibition of tumor growth and prolonged survival. Based on these results, GSC-mediated remodeling of the perivascular niche enables GBM progression. The results also suggest that targeting these GSC-derived vascular pericytes may suppress tumor growth and limit resistance to current antiangiogenic therapies [17]. A reasonable assumption is that CSC-derived pericytes might be in a “transitional state” during CSC differentiation into ECs, but further investigation is required in order to confirm whether there is a relationship between tumor-derived pericytes and ECs [142].

To sum up, there is a bidirectional cross talk between GSCs and perivascular niche: on one hand, perivascular niches enhance stem-like proprieties of GSCs, promote invasion and metastasis of these cells, and promote GSCs escape from therapy. On the other hand, GSCs promote EC migration and angiogenesis and are involved in the recruitment process of endothelial progenitor cells. However, GSCs induce the remodeling of perivascular niches, generating ECs and pericytes and inducing angiogenesis/vasculogenesis. Elucidation of these vascular processes will offer new mechanistic insights into the malignancy of glioblastomas that are commonly characterized by tumor angiogenesis. These findings highlight the complexity of the cellular constituents of glioma neovascularization which should be taken into account in new antiangiogenic strategies for gliomas.

### 3.2. The Hypoxic/Perinecrotic Niche

As a diagnostic hallmark of GBM, hypoxia represents an essential aspect of the glioma microenvironment. Hypoxia promotes tumor angiogenesis, cancer aggression, and therapeutic resistance to various therapies [4]. It also supports GSC self-renewal, proliferation, and tumorigenicity and can induce non-GSCs to acquire GSC features and increased tumorigenic potential [143]. Hypoxia stimulates the expression of the transcription factor, hypoxia-inducible factor (HIF) family. This results in the production of proangiogenic growth factors [107]. Thus, several current publications suggest that the hypoxic niche has a pivotal role in the maintenance and expansion of GSCs [58].

Mediated HIF-1 and HIF-2 represent mediated responses to hypoxia. They comprise a *β* subunit (oxygen-insensitive) and *α* subunit (oxygen-regulated) [144]. Remarkably, HIF-2*α* is particularly involved in the activation of signaling pathways regulating stem cell maintenance [145]. HIF-2*α* is still elevated under chronic hypoxia, whereas HIF-1*α* only gets transiently upregulated [58].

Li et al. were the first to report the involvement of the HIF pathway in GSCs [10]. Using xenograft glioma-initiating,* in vitro* neurosphere formation assays and CD133 expression, they observed significant enhancement of stem cell activity under a hypoxic environment. When either HIF1*α* or HIF2*α* is silenced by shRNA, stem cell activity under both normoxic and hypoxic environments is reduced. Considering HIF2*α* mRNA levels correlate with glioma activity, progression, and prognosis, they highlighted that HIF2*α* is crucial for glioma stem cell activity. Since HIF-1*α* protein levels may be regulated by posttranscriptional mechanisms, this can result in the lack of correlation between HIF1*α* mRNA levels and stem cell activity [146].

GSCs are enriched in perinecrotic regions of human glioblastoma biopsies. They are characterized by reduced oxygen tension and activation of HIF-1*α* and HIF-2*α* [147]. In culture, hypoxia upregulates HIF-1*α* and HIF-2*α* in GSCs. HIF-2*α* is directly involved in promoting the GSC phenotype, whereas HIF-1*α* appears to play a more general, permissive role in GSC maintenance, possibly by enabling cell survival. Furthermore, HIF-1*α* is expressed in both GSCs and non-GSC cells, whereas HIF-2*α* is specifically expressed in GSCs [10, 147]. HIF-2*α* upregulates key genes involved in the induction of a pluripotent state [148], including Klf4 and the direct HIF-2 targets Sox2 and Oct4 [149, 150]. Besides, HIF-2*α* activates c-Myc, another fundamental stem cell regulator, by promoting its interaction with the transcriptional cofactors Sp1, Miz1, and Max [151], suggesting that HIF-2*α* is a key regulator of the undifferentiated phenotype of GSCs in the hypoxic niche.

#### 3.2.1. Soluble Factors

VEGF expression in both GSCs and non-GSCs is induced by hypoxic conditions, but the VEGF levels are constantly higher in GSCs [10]. High-level production of VEGF by GSCs can promote angiogenesis and their tumor-initiating capacity [19]. The upregulation of VEGF signaling as well as promotion of angiogenesis is highly influenced by HIF, resulting in maintenance of the tumor and its microenvironment [152].

Recently, evidence has emerged indicating that antiangiogenic therapies may induce a more invasive phenotype in recurrent tumors [153, 154]. A more hypoxic microenvironment induced by vessel regression is believed to be the important cause of a switch to a more invasive program [155]. In addition, hypoxia leads to enrichment of GSCs, with a more invasive phenotype. Therefore, when exploring new antiangiogenic strategy, how to prune excessive vessels without aggravating hypoxia should be taken into consideration.

**Figure 2:**
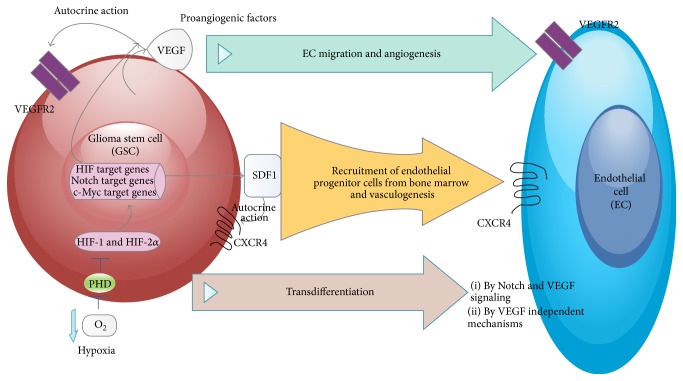
*Effects of hypoxia on GSCs and effects of GSCs on the endothelial cells. GSCs produce proangiogenic growth factors VEGF and HDGF* that stimulate EC migration and angiogenesis;* SDF-1* stimulates recruitment of endothelial progenitor cells from bone marrow and vasculogenesis; GSCs can transdifferentiate under hypoxic condition into ECs.

Recruitment of endothelial and pericyte progenitor cells to promote neovascularization in glioblastoma and regulate the invasion of GBM cells could be induced by HIF1*α*, partly through increases in SDF1*α* [156] (Figure 2).

#### 3.2.2. Cells: Cell to Cell Communication

Since phosphatidylinositol 3-kinase (PI3K)/Akt and ERK1/2 pathways inhibition reduced the fraction of CD133+ GSCs, hypoxia-driven GSC expansion depends on them [157]. Under hypoxic conditions, the Notch pathway is also activated in GSCs [158], through HIF-1*α*. Notch activation led to upregulating the expression of GSC markers such as CD133, Nestin, Bmi1, and Olig2, maintaining GSCs pool and phenotype, and growth of tumor neurospheres and xenografts [147]. Notch inhibition resulted in reduced proliferation and increased apoptosis of GSCs, associated with decreased Akt and STAT3 phosphorylation [159]. HIF-1*α* induced activation of Notch pathway is critical for hypoxia-mediated maintenance of GSC. Either depletion of HIF-1*α* or inactivation of Notch signaling partly inhibits the hypoxia-mediated maintenance of GSCs [160].

Hypoxia upregulates various additional genes involved in the regulation of GSC, such as CXCR4 [157], lysyl oxidase (LOX), hypoxia inducible gene 2 (HIG2) [158], HIF-2 target genes glucose transporter 1 (GLUT1), the proteinase inhibitor Serpin B9, Oct4, and VEGF [10].

GSCs expressed higher levels of histone methyltransferase mixed-lineage leukemia 1 (MLL1) induced by hypoxia than matched nonstem tumor cells, and depletion of MLL1 inhibited HIF transcripts and then reduced the self-renewal, growth, and tumorigenicity of GSCs [161].

When compared to tumors without a mutation, HIF-1*α* levels were higher in human gliomas harboring an IDH1 mutation. Hence, IDH1 seems to work as a tumor suppressor that, when mutationally inactivated, participates to tumorigenesis, partly through induction of the HIF-1 pathway [162].

#### 3.2.3. Metabolic Reprogramming

Hypoxia is also responsible for metabolic reprogramming, leading to acidification of the tumor microenvironment. Acidic conditions promote the expression of GSC markers, self-renewal, and tumor growth in gliomas. GSCs exert paracrine effects on tumor growth through elaboration of angiogenic factors, and low pH conditions increase this expression associated with induction of HIF2*α*. The induction of HIF2*α* and other GSC markers by acidic stress can be reverted by elevating pH* in vitro*, suggesting that raising intratumoral pH may be beneficial for targeting the GSC phenotype. Therefore, when exposing to low pH, it promotes malignancy through the induction of a GSCs phenotype, and culturing cancer cells at lower pH reflective of endogenous tumor conditions may better retain the cellular heterogeneity found in tumors [163, 164].

#### 3.2.4. miRNAs

miRNAs act as critical mediators of hypoxia signaling according to recent studies reported [165]. The pioneering work of Ivan's team demonstrates that a specific set of hypoxia-regulated miRNAs (HRMs) modulates cell cycle, apoptosis, and DNA repair pathways in response to hypoxia in breast cancer [166, 167]. miR-210-3p has been found highly induced in hypoxic glioma cell lines (U87MG and U251MG) and in hypoxic GBM tumor samples, pointing to its use as a hypoxia marker or therapeutic target in GBM. Several studies have since then found that HRMs fine-tune their hypoxic response through cellular mechanisms, such as angiogenesis, cell cycle regulation, metabolism, apoptosis, metastasis, proliferation, and resistance to anticancer therapy [168, 169]. GBM survival in the tumor microenvironment is promoted by miR-210-3p, along with aggressiveness by imparting temozolomide resistance and targets HIF3A, which is known to function as a negative regulator of hypoxia-inducible gene expression [170]. Agrawal et al. point to miR-210-3p as an oncogenic player and a novel potential intrinsic marker of hypoxia in glioblastoma [171].

In conclusion, a crucial regulatory role for the GSC phenotype is played by the hypoxic microenvironment, by directly inducing the expression of self-renewal genes, suppressing differentiation, and promoting the cross talk between HIFs and other signaling pathways required for GSC maintenance.

These discoveries emphasize the key role of the microenvironment in regulating the differentiation status of tumor cells and its possible involvement in controlling the plasticity of the cancer stem cell hierarchy.

### 3.3. The Immune Niche

The immune system appears to have a central role in the control of tumor progression [172]. Recent studies show a direct interaction of GSCs with immune cells, highlighting the major role of these components in the GSCs niche. Furthermore, GSCs and inflammatory cells are involved in a dynamic cross talk involving GSC-mediated induction of immune cell infiltration, generation of a protumorigenic inflammatory environment, and inflammation-driven cancer promotion [58].

Tumor-associated macrophages (TAMs) represent prevalent tumor-infiltrating inflammatory cells in GBM [173, 174]. The great number of TAMs in GSCs niche suggests their key role in GBM tumor progression, also positively correlated with the malignancy grade [175]. TAMs are mainly located near CD133+ GSCs, around microvessels [176] and in hypoxic areas [177], suggesting a direct interaction between GSCs and TAMs. Enhanced expression of proinflammatory genes like RAGE, COX2, and NF-*κ*B was recently found in hypoxic niche of GSCs [178]. When compared to differentiated tumor cells, the GSCs show an increased capacity in active chemoattraction and recruitment of TAMs, processes mediated by chemokines and growth factors, secreted by GSCs, including VEGF, neurotensin, SDF1, and soluble colony-stimulating factor 1 (sCSF-1) [177, 179]. GSCs also secrete factors that support the growth of macrophages and induce the polarization of TAMs into the immunosuppressive M2 phenotype [68].

Although the above-mentioned molecules prove the important roles of GSCs in immune cell modulation, leading to the induction of tumor promoting inflammation, understanding the impact of immune cells on GSC maintenance is still limited. Molecules/cytokines like TGF*β*, VEGF, SDF1, bFGF, and NO produced by immune cells [180–182] have been independently proven to maintain and promote GSCs [58], pointing to speculate that the protumorigenic function of specific sets of inflammatory cells is additionally mediated through the direct stimulation of GSCs, which will most certainly represent a motivating area of research in further studies.

The origin of TAMs, whether they are recruited from locally activated microglia or from the peripheral monocyte population, represents an important question to be answered, with high addressability to potential future therapy targets. Oncogenically, microglia are the only resident macrophages that are known to be exclusively derived from yolk sac macrophages without monocyte intermediates. To maintain their population, they rely on local proliferation [183]. Recent studies have identified a unique microglial subpopulation as an indispensable component of the subventricular neurogenetic zone and rostral migratory stream, establishing the framework for a preexisting collaborative cross talk between brain resident immune and stem cells [184].

Over two decades ago, the relationship between microglia and brain tumors has been first mentioned [175, 185], when they were identified as the “Achilles heel” of the immune system [186], exerting a surprisingly low cytotoxic activity.* In vitro* long-term cocultures of glioma and microglial cells showed a short activation of phagocytic properties, followed by a steady state depression.

In 2015, Zhou et al. provide new insights into where and how TAMs are recruited and educated by GSCs in GBMs [187]. They have analyzed the molecular relationship between GSCs and TAM recruitment in GBMs and demonstrated that GSCs secrete periostin (POSTN), a GSC-secreted cytokine, to potently attract and recruit peripheral monocytes. The correlation between TAM density and glioma grade points to a supportive role for TAMs in tumor progression, which can be altered by POSTN-directed blockade.

Inhibition of macrophage-colony stimulation factor receptor demonstrated myeloid compartment involvement in glioma initiation and progression [188]. The penetration of vascular borne cells into tumoral tissue offers vehicle for peripheral delivery of cytotoxic drugs, in an otherwise protected environment. Apart from blood brain barrier diffusibility, therapeutic monocytes have the advantage of tissue depth diffusion, almost three times higher than nanoparticles [189].

Accumulated knowledge on TAMs and GSCs roles on immune cell modulation, possibly delineating a third niche, underlined the role of immune system in GBM progression and GSCs escape from therapy.

## 4. Challenges in Targeting the Tumor Microenvironment

Cancer tissue does not represent a homogenous population of clonally expanded cancer cells; this constitutes an important paradigm shift in cancer research [190]. GSCs have a high capacity for self-renewal and tumorigenic potential [191].

Since conventional therapeutic approaches have not been developed to target GSCs, many such cells are enriched by conventional cancer therapy [35]. The unsuccessful removal of GSCs constitutes an important reason for which cancer relapse follows conventional therapy and, thus, it is a major obstacle to efficient cancer treatment [192]. Cancer cells and host cells form a tumor microenvironment that allows tumor initiation and progression. Because conventional cancer therapy approaches have been developed without emphasizing the tumor microenvironment, a key new focus for cancer therapy is to limit cancer development by targeting GSCs microenvironment: perivascular niche, hypoxic niche, and immune evasion [193].

Interrupting the perivascular niche might prove a critical approach for GSCs targeting. Directly targeting endothelial cells that constitute the tumor vasculature is an alternative approach that has been used to destabilize GSCs function. Thus, a modification of this microenvironment can decrease GSCs tumorigenicity. One approach is the use of antiangiogenic drugs, which decrease blood flow toward the tumor and induce local alterations to finally decrease the number of GSCs or render them sensitive to other therapies [194]. The monoclonal antibody bevacizumab (Avastin) and the novel small molecule pan-VEGF inhibitor cediranib (AZD2171) that targets VEGF (in order to disrupt VEGF/VEGFR interaction) are currently used in clinical therapy somewhat successfully. Treating mice bearing GSCs initiated xenografts with bevacizumab or other antiangiogenic agents (e.g., anti-SDF1 drug AMD3100) delayed tumor growth* in vivo* due in part to a decrease in tumor blood vessels as well as the percentage of GSCs [195]. Considering the use of antiangiogenic drugs has become widespread, it has been found that tumors develop mechanisms of resistance to antiangiogenic drugs. Recent studies have raised concerns that system anti-VEGF treatment may improve short-term patient outcome but may ultimately lead to more aggressive malignancies. By “pruning” leaky vessels, anti-VEGF drugs like bevacizumab may in fact improve overall tumor vasculature. This could lead to increased invasion and more aggressive growth [196]. Combining antiangiogenic treatment with cytotoxic agents leads to xenografted glioma tumors depleted of GSCs and reduced in size after treatment [197].

IL-6 is another secreted factor known to support angiogenesis, which can be produced by the tumor microenvironment. Higher levels of IL-6 mRNA are directly linked to poor patient survival in GBM. IL-6 receptors, gp130 and IL-6R*α*, are preferentially expressed on GSCs and their expression can be stimulated by hypoxia, important component of the tumor microenvironment. Directly targeting IL-6 or IL-6R*α* by shRNA impairs GSC growth and survival* in vitro*, suggesting the significance of IL-6 autocrine signals in GSC maintenance [50]. Notably, administration of anti-IL-6 antibody delayed the growth of tumors initiated with GSCs, suggesting that targeting IL-6 may be useful as antiglioma therapies [196].

Angiogenesis, cell migration, and tumor resistance are induced by hypoxic microenvironment. Therefore, HIFs constitute an important molecular target to be developed for novel therapeutic strategies in order to inhibit GBM malignant progression [198]. An increasing number of chemical compounds have been shown to inhibit HIF activity through a wide variety of molecular processes and to counteract tumor growth in GBM xenograft models. There are several molecular effects driven by these compounds, such as decreased levels of HIF-1*α*, mRNA, and protein synthesis, inhibition of HIF-*α* heterodimerization with ARNT, block of HIFs binding to DNA and decrease of its transcriptional activity, and increased HIF-1*α* degradation. Even though the number of inhibitor molecules of HIF-1 has rapidly increased lately, only few compounds are progressing towards preclinical and early clinical development. Remarkably, the combination of HIF-1 inhibitors with existing treatments or new-targeted therapies could prove useful in the clinical practice [199].

It appears that inflammation may be influenced by HIF, including the adaptive and innate inflammatory responses [200–202]. The shared requirement for HIF in GSCs and inflammatory cells raised the interesting prospect that GSCs and inflammation, two important challenges in cancer therapy, may be addressed by targeting HIF [146].

The hypoxic niche and HIF-1*α* have been reported to enhance the migration properties of GSCs by promoting metalloproteinase expression and migration-associated receptors, such as CXCR4 [203]. These data are supported by the hypothesis that most hypoxic cells could migrate through the above-described layers [204], potentially invade normal brain tissues, or maintain the GSC population of the most peripheral layers. For this reason, the migratory features of GBM cells could prove to be a valid therapeutic target for this tumor.

The key chemokine that has been associated with the migratory process of GBM is the SDF-1/CXCL12. The activated CXCR4/CXCL12 complex is rapidly internalized from the cell surface. GBM cells are endowed with a high expression of CXCR4. Moreover, a strict correlation between CXCR4 levels and the infiltrative extension of GBM tumors exists. Recently, it has been postulated that this receptor might be a cell surface marker for GSCs [129]. Another process by which the CXCR4/CXCL12 axis contributes to GBM growth is its ability to recruit endothelial and marrow cells to support tumor vasculogenesis and angiogenesis [205]. In this context, plerixafor (AMD3100) is a bicyclam molecule that antagonizes the binding of SDF-1 to CXCR4 and inhibits irradiation-induced vasculogenesis* in vivo* [195].

Hypoxic stimuli are not the same all over tumor mass and different zones described for each GBM have been mentioned recently. Genome, transcriptome, and methylome analysis of different areas of the same tumor (necrotic zone, tumor zone, interface, and peripheral brain zone) revealed that “transcriptome heterogeneity was much more important within tumors than between patients.” Tumor subtype, as assessed by 840 gene signatures, differed between the aforementioned zones: the neural and proneural subtypes were located in peripheral brain zone and interface, whereas mesenchymal and classical subtypes were found in tumor and necrotic zones [206]. These results would imply that aggressive subtypes are peripherally located, favoring local invasion into healthy tissue. This brings into focus a new microenvironment, the peripheral brain zone, which harbors 90% of tumor recurrences [207]. This area includes, apart from infiltrated aggressive tumor cells, reactive astrocytes, inflammatory cells, and “glioblastoma-associated stromal cells.” These stromal cells are diploid, share phenotypic and functional properties with cancer-associated fibroblasts, and do not recapitulate the genomic alterations typical of tumor cells [208].

Immune cell recruiting into tumoral zone was viewed as a “Trojan horse” for cytotoxic drug delivery. The major problem to overcome was the cytotoxicity of the load that would affect the very cargo that carries it. The alternative to overcome this problem is loading with nanoparticles for photothermal therapy [209] or increase delivery through opening the blood brain barrier [210].

To conclude, the discoveries of all these studies suggest that both the hypoxic and the perivascular niche could prove an efficient target for GSCs treatment. Understanding the biological behavior of GSCs, their regulatory processes and their niches may directly impact current efforts for directed therapeutics against the highly aggressive gliomas [152]. Consequently, multimodal therapies that include combinations of antiangiogenic therapies along with cytotoxic therapies should be able to overcome this problem. The simple eradication of the existing GSCs is not enough to provide a cure for gliomas; however, obstructing the potential sources of GSCs as well as ameliorating the local tumor inducing/promoting microenvironment represents a reasonable strategy [211].

## 5. Conclusions

There is an urgent need for understanding the cross talk between GSCs and their niches, which supports the GSCs self-renewal, tumor invasion, and metastasis, as well as GSCs escape from therapy. Although many questions and controversies remain, the progress has been driven by the interest in the microenvironment that induces particular signatures in order to regulate GSCs maintenance and function. Novel therapeutical approaches should disrupt the protective niches, perivascular, hypoxic, and immune, of GSCs, in order to improve and even to revolutionize current diagnosis and therapy of gliomas. Therefore, effective control of the GSCs microenvironment will likely complement the conventional approach of cancer therapy, aiming at eradicating GSCs.

## Figures and Tables

**Table 1:** Key molecules involved in normal neural stem cells and in glioma cancer stem cells.

Name	Roles/involvement	Reference(s)
Receptors		
Notch-1	Notch signaling enhances NSC survival, proliferation, and self-renewal during embryonic CNS development.	[43]
Notch-2	Primary GSCs have high Notch-2 expression. Constitutive Notch-2 signaling in neural stem cells generates similar features to GSCs. Notch signaling represents a pathway common in the midst of regulating the GSC phenotype.	[44, 45]
PTCH1 (protein patched homologue 1)	Proliferation of NSC and GSC. PTCH highly expressed in astrocytoma, oligodendroglioma, and GBM.	[46]
PROM1/CD133 (prominin-1)	Maintenance of stem-cell properties (differentiation suppressor) lost during CSC differentiation, different glycosylation pattern in CSC.	[22]
CXCR4	Stimulates proliferation and promotes GSC-mediated angiogenesis.	[47]
EGFR (epidermal growth factor receptor)	Often amplified and mutated in high-grade gliomas. Increases proliferation and tumorigenicity, inhibits apoptosis, regulates angiogenesis and stemness, and mediates resistance to oxidative stress and ionizing radiation.	[48, 49]
IL-6R*α*	Promotes self-renewal, GSC maintenance, and tumorigenicity and suppresses apoptosis.	[50]
Integrin *α*6	Promotes self-renewal, proliferation, tumorigenicity, and GSC marker.	[25]
PDGFRA (platelet-derived growth factor receptor-alpha protein)	Conversion of oligodendrocyte progenitors into neural stem-like cells. Expressed in gliomas. Amplified and mutated in glioblastoma.	[51]

Ligands		
BMPs (bone morphogenetic proteins)	Reduce proliferation and abolish tumorigenicity. Induce differentiation, determined by an increase in the number of glial fibrillary acidic protein- (GFAP-) positive cells and delay tumor growth.	[52, 53]
SHH (sonic hedgehog protein)	In GSCs, Hedgehog-Gli signaling increases expression of stem genes (e.g., CD133, Olig2, Oct4, Nanog, and Sox2), promotes self-renewal, and supports glioma growth and survival. Proliferation of NSC and GSC. Activation of SHH pathway in brainstem glioma.	[54–57]
TGF*β*	Promotes self-renewal, tumorigenicity, proliferation, and invasion and maintains stemness in GSCs.	[58, 59]
WNT	Regulates GSCs maintenance, proliferation, and tumorigenicity, inhibits apoptosis and differentiation, and regulates cell migration.	[60]

Transcription factors and chromatin-modifying proteins		
Bmi1 (polycomb complex protein)	Found in undifferentiated NSCs and high grade gliomas, with higher expression correlating to poor glioma patient survival. Found enriched in GSCs and required for their self-renewal.	[61, 62]
Oct4	Oct4 is highly expressed in human gliomas and correlates with tumor grade, promotes colony formation, and inhibits differentiation in glioma cells, potentially through upregulation of phosphorylated STAT3.	[59]
Sox2	Oct4 and Sox2 are increased in GSCs and promote tumorigenic activity as validated by tumor sphere formation and intracerebral tumor formation.	[29, 32]
Nanog	Nanog expression is higher in GSCs, coexpressed with CD133+ glioma cells and less expressed in regions enriched for the differentiation marker, GFAP. Interacts with the Hedgehog-Gli pathway to modulate GSC proliferation, neurosphere formation, and tumor promotion in orthotopic xenografts.	[31, 63]
c-Myc	c-Myc levels correlate with glioma tumor grade and are highly expressed in GSCs relative to non-GSCs. Not only does c-Myc promote proliferation, but it may also represent a GSC-specific survival factor. c-Myc is highly expressed in approximately half of CD133+ cells acutely isolated from primary human GBM specimens, whereas c-Myc expression is considerably lower in the CD133− fraction.	[64, 65]
Olig2	Highly expressed in diffuse gliomas including astrocytomas, oligodendrogliomas, and oligoastrocytomas. Controls GSC proliferation, cell adhesion, and cell cycle progression.	[66, 67]
STAT3	Promotes proliferation, stemness, self-renewal, tumorigenicity, immunosuppression, induction of Tregs, and TAMs and inhibits apoptosis.	[68–70]
Musashi (RNA-binding protein Musashi homolog 1)	Protein alteration favors tumorigenesis. NSC and HSC display Musashi alteration. Consistently correlated with the tumor proliferation in gliomas. Expressed in GSCs and in neurospheres derived from brain tumors, being correlated with tumor grade and proliferation rate.	[71]
GLI1, GLI2, and GLI3	GLI1 protein expressed in NSC. Originally isolated from glioblastoma. GLI expressed in GBM, astrocytoma, and oligodendroglioma.	[46]

miRNAs underexpressed		
miR-7	Inhibits GSCs proliferation and invasion.	[72]
miR-124 and miR-137	Decrease proliferation and increase differentiation of GSCs; G1 arrest.	[73, 74]
miR-34a	Inhibition of invasion, proliferation, and cell cycle progression; inhibition of Notch-1, Notch-2, and c-Met.	[75]
miR-451 and miR-452	Decrease proliferation and viability.	[76]
miR-101	Decreases invasion, proliferation, and angiogenesis.	[77]
miR-218	Decreases migration, proliferation, and self-renewal.	[78]
miR-451	Decreases proliferation and viability and inhibits self-renewal.	[79]

miRNAs overexpressed		
miR-21	Increases proliferation and invasion and decreases apoptosis and chemoresistance.	[72, 80, 81]
miR-10b	Increases invasiveness.	[82]
miR-17-92 cluster	Increases tumorigenesis and cell cycle progression.	[83]
miR-93	Increases tumor growth and angiogenesis.	[84]
miR-125b	Decreases apoptosis.	[85, 86]
miR-196a	Decreases patient survival.	[87]

Enzyme		
IDH1/IDH2** (**isocitrate dehydrogenase 1/2)	Catalyses neomorphic formation of 2-hydroxyglutarate. Frequent in astrocytomas, oligodendrogliomas, and glioblastomas.	[88]

Intermediate filament		
Nestin	Frequently expressed in high grade gliomas, especially in primary tumors in patients with dissemination, being a predictive marker for poor survival rate. Positive coexpression for Nestin/CD133 is an indicator of poor prognosis.	[28, 89]
